# Defining Biological Networks for Noise Buffering and Signaling Sensitivity Using Approximate Bayesian Computation

**DOI:** 10.1155/2014/625754

**Published:** 2014-06-05

**Authors:** Shuqiang Wang, Yanyan Shen, Changhong Shi, Tao Wang, Zhiming Wei, Hanxiong Li

**Affiliations:** ^1^Shenzhen Institutes of Advanced Technology, Chinese Academy of Sciences, Shenzhen 518000, China; ^2^Department of Orthopaedics and Traumatology, University of Hong Kong, Hong Kong; ^3^Department of Mechanical and Biomedical Engineering, City University of Hong Kong, Hong Kong; ^4^School of Public Health, Guangzhou Medical University, Guangzhou 510000, China; ^5^Specialist Union Co., Ltd, Shenzhen 518000, China; ^6^Shandong Academy of Agricultural Machinery Sciences, Jinan 250000, China; ^7^Department of Systems Engineering and Engineering Management, City University of Hong Kong, Hong Kong

## Abstract

Reliable information processing in cells requires high sensitivity to changes in the input signal but low sensitivity to random fluctuations in the transmitted signal. There are often many alternative biological circuits qualifying for this biological function. Distinguishing theses biological models and finding the most suitable one are essential, as such model ranking, by experimental evidence, will help to judge the support of the working hypotheses forming each model. Here, we employ the approximate Bayesian computation (ABC) method based on sequential Monte Carlo (SMC) to search for biological circuits that can maintain signaling sensitivity while minimizing noise propagation, focusing on cases where the noise is characterized by rapid fluctuations. By systematically analyzing three-component circuits, we rank these biological circuits and identify three-basic-biological-motif buffering noise while maintaining sensitivity to long-term changes in input signals. We discuss in detail a particular implementation in control of nutrient homeostasis in yeast. The principal component analysis of the posterior provides insight into the nature of the reaction between nodes.

## 1. Introduction

A challenge in systems biology is to understand the mechanism by which cells sense and process external information using biochemical networks of interacting genes and proteins. The precise nature of information flow through a biological network, which is governed by factors such as response sensitivities and noise buffering, greatly affects the operation of biological systems. Quantitative analysis of these properties is often difficult in naturally occurring systems but can be greatly facilitated by studying simple synthetic networks [1–3]. Generally, labeling “noise” or “signal” depends on the object's function. There exist a lot of cases where noise is not a negative component for the system, but it adds dynamical behavior such as the case of the bursts in gene expression [4]. The “noise” in this work refers to the object that can weaken wanted signal. Reliable information processing requires high sensitivity to changes in the input signal but low sensitivity to random fluctuations in the transmitted signal. Recent studies have shown that linear cascades display an interplay between sensitivity to changes in input signal and the ability to buffer stochastic fluctuations [5–7]. A key question is whether network connectivity, for example, the presence of positive or negative feedbacks, can modulate this interplay, reducing propagated noise while maintaining high sensitivity. Dublanche et al. revealed the negative feedback loop as being a way to control and decrease transcriptional noise via experiment [8]. Becskel and Serrano and Simpson et al. also argued that negative feedbacks can buffer noise relative to linear cascades [9, 10]. These studies, however, did not consider the associated changes in signaling sensitivity. Hornung and Barkai argued that negative feedback can buffer noise, but this buffering comes at the expense of an even greater reduction in signaling sensitivity [3]. Guantes et al. [11] introduced an analytical framework to study the amplitude and frequency response of a general class of two-component genetic circuits and showed that the presence of a feedback interaction in the detection module imposes a trade-off on amplitude and frequency detection, whose intensity depends on feedback strength. They also observed that coherent feed-forward loops can act as good frequency and amplitude noise-tolerant detectors [11]. Based on the framework of Hornung and Barkai's handling issues, we employ the approximate Bayesian computation scheme to select the biochemical circuits that can buffer propagated noise while maintaining signaling sensitivity. Our approach allows us to exploit methods from Bayesian statistics, including efficient exploration of models spaces and high-dimensional parameter spaces and the ability to rank models with respect to their ability to maintain signaling sensitivity while minimizing noise propagation.

Gene expression is a stochastic or noisy process. This noise comes about in two ways [12]. The inherent stochasticity of biochemical processes such as transcription and translation generates “intrinsic” noise. In addition, fluctuations in the amounts or states of other cellular components lead indirectly to variation in the expression of a particular gene and thus represent “extrinsic” noise. In the current work, we mainly focus on the case that the sole noise source is fluctuations in the input node, namely, the “extrinsic” noise. In the current study, the signal is defined as a long-term change in the input, while the noise is characterized by rapid stochastic fluctuation. Both signal and noise can be described as time series, and the difference is mainly in the time scale of changes.

Typically, there are often many possible alternative network circuits that are capable of executing a particular biological function. Some topologies may be more favorable because of robustness. Distinguishing models and finding the most suitable ones are an important challenge in systems biology, as such model ranking, by experimental evidence, will help to judge the support of the working hypotheses forming each model. However, we often lack reliable information about model parameters and the likelihood surfaces of large models are complex. Here, we employ a novel statistical tool, approximate Bayesian computation scheme, that allows us to (i) compare the performance of different models and (ii) estimate the posterior distributions of model parameters. Approximate Bayesian computation (ABC) is a popular approach to address inference problems, where the likelihood function is intractable or expensive to calculate [13–17]. In ABC methods, the evaluation of the likelihood is replaced by a simulation-based procedure [18–22]. Despite representing a substantial methodological advance, existing methods based on rejection sampling or Markov chain Monte Carlo can be inefficient and accordingly require more iterations than may be practical to implement. Recently, the ABC method based on sequential Monte Carlo (SMC) is suggested to overcome these inefficiencies [23–26]. In this work, to design the most favorable biological circuits that can effectively buffer propagated noise while maintaining high signaling sensitivity, we utilize the ABC method based on sequential Monte Carlo to rank model structures and design the corresponding model parameters.

## 2. Models and Methods

### 2.1. Models

**Figure 1:**
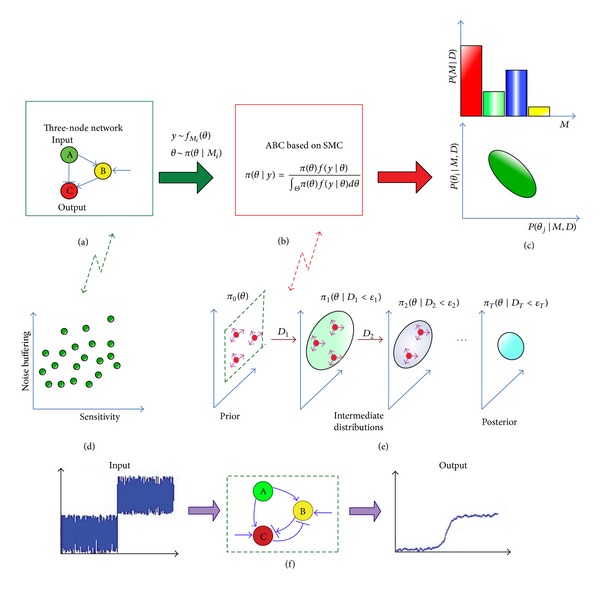
The framework of the proposed method. (a) A three-node candidate network with “⊣” indicating negative regulation and “→” indicating positive regulation. (b) The employed model selection tool. (c) The model posterior probability indicates the ability of each network structure to buffer noise without losing sensitivity. The parameter posterior shows parameters that are sensitive or insensitive to the input-output specification. (d) The noise buffering and sensitivity are employed to specify the input-output character of the three-node biological network. (e) The model parameter is evolved using sequential Monte Carlo. (f) A typical profile of an input signal and a profile of the output signal after going through a three-node network that can maintain signaling sensitivity while minimizing noise propagation.

To analyze the effect of network architecture on the interplay between noise buffering and sensitivity, we consider the network that is composed of three nodes, one input node, *n*_A_, and two other components, *n*_B_ and *n*_C_, as shown in Figure 1(a). Generally, we define the node, *n*_C_, as the output node. Based on the Fluctuation Dissipation Theorem [27–29], the system of equations that describe the kinetic response of the network is given by

(1) $$\begin{array}{c} \frac{dn_{i}}{dt}=J_{i}^{+}\left(n_{\text{A}},n_{\text{B}},n_{\text{C}}\right)-J_{i}^{-}\left(n_{\text{A}},n_{\text{B}},n_{\text{C}}\right), \end{array}$$

where *J*_*i*_^+^ and *J*_*i*_^−^ are the total fluxes of production and elimination of *n*_*i*_ and they are determined by Michaelis-Menten constants and *i* = A, B, C.

At a steady state the equations given in (1) equal zero; hence,

(2) $$\begin{array}{c} \langle J_{i}^{+}\rangle =\langle J_{i}^{-}\rangle =\langle J_{i}\rangle . \end{array}$$

The triangular brackets denote the steady-state average.

### 2.2. Methods

ABC methods have been conceived with the aim of inferring posterior distributions, where likelihood functions are computationally intractable or too costly to evaluate. They exploit the computational efficiency of modern simulation techniques by replacing the calculation of the likelihood with a comparison between the observed and simulated data. The disadvantage of the ABC rejection sampler is that the acceptance rate is low when the prior distribution is very different from the posterior distribution. To avoid this problem, the ABC algorithms based on SMC have been developed [22, 23]. In ABC SMC, a number of sampled parameter values (called particles), {*θ*_1_, *θ*_2_,…, *θ*_*n*_}, sampled from the prior distribution *π*(*θ*), are propagated through a sequence of intermediate distributions, *p*(*θ* | *D*_*t*_ < *ε*_*t*_), *t* = 1, 2, …, *T* − 1, until it represents a sample from the target distribution *p*(*θ* | *D*_*T*_ < *ε*_*T*_) with *T* indicating the final state, as shown in Figure 1(e); here *D* relates model output to the desired output characteristic. In Bayesian inference, comparison of a discrete set of models can be performed using the marginal posterior. We define *M* as model space and *θ* as the parameter space. Considering the joint space defined by {*M*, *θ*} ∈ *M* × Θ_*M*_, Bayes theorem can then be written as [24]

(3) p(M ∣ y)=p(y ∣ M)π(M)∫Mp(y ∣ M′)π(M′)dM′=p(y ∣ M)π(M)∑Mp(y ∣ M′)π(M′),

where *p*(*y* | *M*) is the marginal likelihood. Therefore, model selection can be incorporated into the ABC framework by introducing the model indicator *M* and proceeding with inference on the joint space {*M*, *θ*}.

To capture the desired output characteristics, the distance function *d*(*y*, y*) is introduced to specify how the model output, *y*, approximates the desired output, y*. In biological model design, we would rarely insist on achieving the true posterior distribution but would like to achieve the objective within some tolerance *ε*_*T*_ [23, 24]. To select the network structures that can buffer propagated noise while maintaining high signaling sensitivity, we introduce two-component distance metric: {*N*, *S*}, where *N* and *S* are the noise buffering and sensitivity, respectively, as shown in Figure 1(d). Since the noise amplification in a linear (unbranched) cascade is precisely proportional to the sensitivity [3, 5], we take this unbranched cascade for comparison with the biological network designed. After that, the distance function can be given by

(4) $$\begin{array}{c} d\left(y,y^{*}\right)=\left\{\frac{N_{U}}{N_{j}},\frac{S_{U}}{S_{j}}\right\}, \end{array}$$

where *N*_*U*_ and *S*_*U*_ are the noise buffering and sensitivity for linear cascade network, respectively, while *N*_*j*_ and *S*_*j*_ are for the biological network designed.

According to the works by Hornung and Barkai [3] and Paulsson [27], we introduce two measures to analyze the interplay between the sensitivity and the noise buffering. The steady-state sensitivity, *s*_*j*_, of component *j*  (*j* = B, C) to changes in the input *n*_*A*_ is given by

(5) $$\begin{array}{c} s_{j}=\frac{\langle n_{\text{A}}\rangle }{\langle n_{j}\rangle }\frac{d\langle n_{j}\rangle }{d\langle n_{\text{A}}\rangle }=\frac{d\ln\langle n_{j}\rangle }{d\ln\langle n_{\text{A}}\rangle }, \end{array}$$

with all quantities measured at steady state. For node *j*  (*j* = B, C), the measure for noise buffering *N*_*j*_ is defined as the ratio between the output and input noise:

(6) $$\begin{array}{c} N_{j}=\frac{\eta_{j}}{\eta_{\text{A}}}=\frac{\text{std}\left(n_{j}\right)/\langle n_{j}\rangle }{\text{std}\left(n_{\text{A}}\right)/\langle n_{\text{A}}\rangle }. \end{array}$$

As above, all quantities are measured at steady state. Both *S* and *N* depend on the different parameters of the system, such as the Michaelis-Menten constants. Following the formalism presented by Paulsson [27], we employed the Fluctuation Dissipation Theorem to derive an analytical formula for sensitivity and noise buffering. Differentiating (1) at the steady state with respect to *n*_A_ and then multiplying by 〈*n*_A_〉/〈*J*_*i*_〉, we obtain

(7) 〈nA〉〈Ji〉(∂〈Ji+〉∂〈nA〉−∂〈Ji−〉∂〈nA〉) +〈nB〉〈Ji〉(∂〈Ji+〉∂〈nB〉−∂〈Ji−〉∂〈nA〉)〈nA〉〈nB〉d〈nB〉d〈nA〉 +〈nC〉〈Ji〉(∂〈Ji+〉∂〈nC〉−∂〈Ji−〉∂〈nC〉)〈nA〉〈nC〉d〈nC〉d〈nA〉=0.

Using (2), we obtain

(8) $$\begin{array}{c} H_{i\text{A}}+H_{i\text{B}}s_{\text{B}}+H_{i\text{C}}s_{\text{C}}=0, \end{array}$$

where the *H*_*ij*_ (*i*, *j* = A, B, C) terms are the reaction flux elasticities [25, 26]:

(9) $$\begin{array}{c} H_{ij}=\frac{\partial \ln\langle J_{i}^{-}\rangle }{\partial \ln n_{j}}-\frac{\partial \ln\langle J_{i}^{+}\rangle }{\partial \ln n_{j}}. \end{array}$$

The elasticity, *H*_*ij*_, is used to measure how the balance between production and elimination of *n*_*i*_ is affected by *n*_*j*_. These scale-free parameters are closely related to the elasticities of metabolic control analysis and the apparent kinetic orders of biochemical systems theory [27]. With the definition of elasticity, the sensitivity of the output can be given by [3]

(10) $$\begin{array}{c} s_{\text{C}}=\left|\frac{H_{\text{BA}}H_{\text{CB}}-H_{\text{BB}}H_{\text{CA}}}{H_{\text{BB}}H_{\text{CC}}-H_{\text{BC}}H_{\text{CB}}}\right|. \end{array}$$

The absolute value facilitates a comparison between systems that increase or decrease their output, when the input goes up. Noise amplification can be found by solving the matrix equation [3, 28]:

(11) $$\begin{array}{c} M\eta +\eta M^{T}+E=0, \end{array}$$

where the matrix *η* is composed of the normalized noise terms and *M* represents a 3 × 3 matrix. The matrix *E* is related to the elasticities and time scales, and the matrix *E* contains a single term corresponding to noise input from *n*_A_. In the construction of *E*, we assume that the sole noise source is fluctuations in *n*_A_. The framework of the proposed method is given in Figure 1.

Note that the stability of a system is a prerequisite in our method for model selection. Now that we have introduced the reaction flux elasticity, *H*, the criteria for stability of the three-node network can be given by [3]

(12) $$\begin{array}{c} \frac{H_{\text{BB}}}{a_{1}}+\frac{H_{\text{CC}}}{a_{2}}>0,\\ H_{\text{BB}}H_{\text{CC}}+H_{\text{BC}}H_{\text{CB}}>0, \end{array}$$

with the *a*_1_ and *a*_2_ terms defined as the degradation time scales for components B and C, respectively.

## 3. Results and Discussions

**Figure 2:**
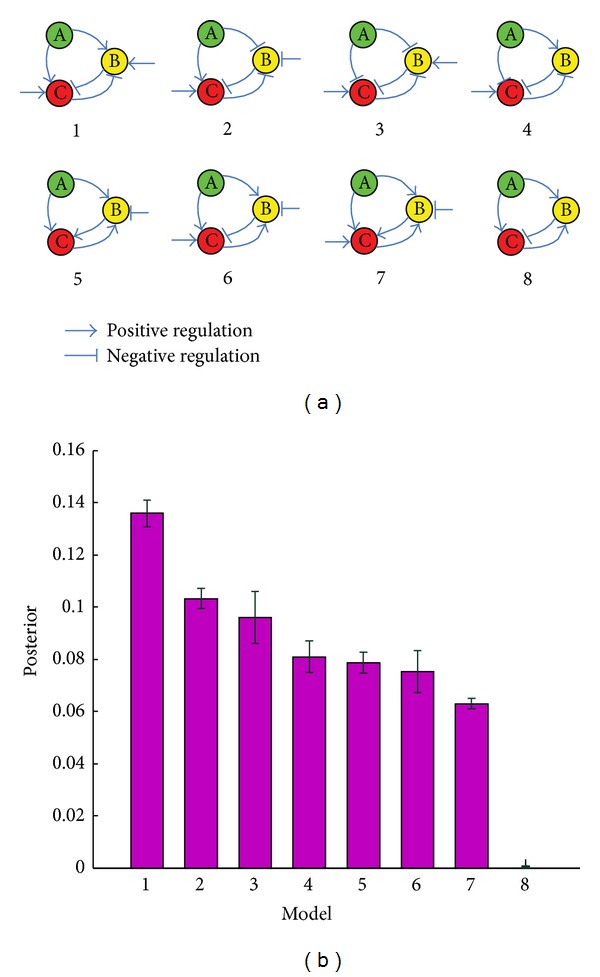
(a) 1–7: the top seven biological circuits. 8: a biological circuit ranked bottom. (b) Posterior probability for buffering noise while maintaining sensitivity. The error bars indicate the variability in the marginal model posteriors over 10 separate runs.

In this work, we require a connection from *n*_B_ to *n*_C_. This connection can either be repressing or activating. There are five additional possible connections in the network (A→B, A→C, B→C, B→B, and C→C) which can be repressing, activating, or nonexisting. Thus we study 2 × 3^5^ − 2 × 3^3^ = 432 specific biological circuits. The prior distributions for elasticity, *H*, and degradation time scale, *a*, are taken to be uniform, and the perturbation kernels for both parameters are uniform, *K*_*t*_ = *σU*(−1,1), with *σ* = 0.2. The number of particles in each population is 1500. We ranked the 432 biological circuits based on their abilities to buffer noise while maintaining sensitivity. The top 7 network architectures are sequentially shown in Figure 2(a). The posterior probabilities of these models are given in Figure 2(b).

**Figure 3:**
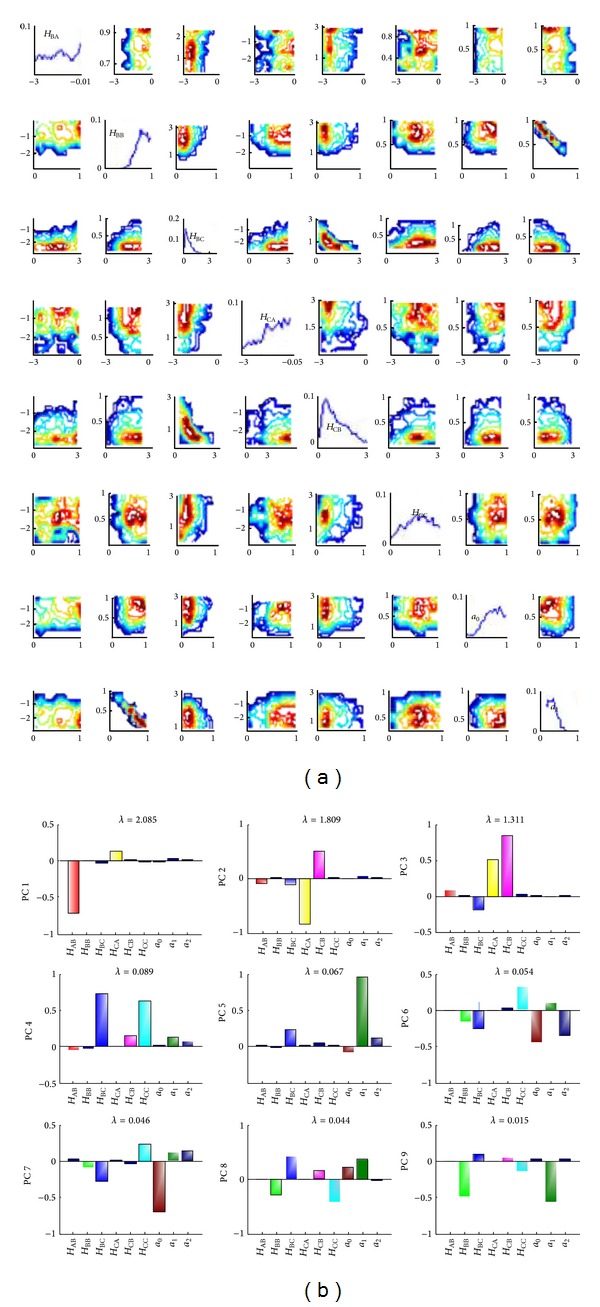
(a) Parameter posterior distribution for biological circuit 1. *a*_0_ and *a*_1_ are the normalized degradation time scales for components A and B, respectively. (b) Principal component analysis of the posterior distribution for model 1. PC1 is the first principal component (accounting for as much of the variability in the data as possible), while PC9 describes the direction of least variance and therefore the most sensitive parameters. *a*_0_, *a*_1_, and *a*_2_ are the degradation constants for components A, B, and C, respectively. *λ* indicates the eigenvalue of variance covariance matrix.

The posterior distribution shows which parameters are correlated. The posterior for model 1 is shown in Figure 3(a). The posterior shows in particular that the self-reaction flux elasticity, *H*_BB_, and the degradation time scale, *a*_1_, are anticorrelated. A principal component analysis (PCA) of the posterior (Figure 3(b)) shows other correlated parameters on the first few principal components. The last principal component indicates the direction of least variance, also the most sensitive parameters. From Figure 3(b) we can deduce that the reaction between node A and node C is less important.

**Figure 4:**
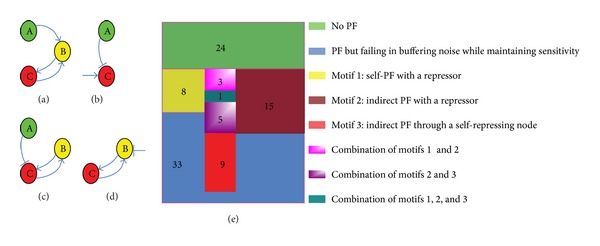
(a) A biological circuit that is not competent for buffering noise while maintaining sensitivity. (b) Motif 1: self-positive feedback with a repressor. (c) Motif 2: indirect positive feedback with a repressor. (d) Motif 3: indirect positive feedback though a self-repressing node. (e) Venn diagram of biological circuits with different characters. PF is short for positive feedback.

Hornung and Barkai used to study noise propagation and signaling sensitivity in biological networks and they highlighted the role of positive feedback for buffering propagated noise while maintaining sensitivity [3]. Our analysis shows that the positive feedback motif in a biological circuit is just a necessary condition for buffering propagated noise while maintaining sensitivity, not the sufficient and necessary condition. We analyzed 81 biological circuits that contain positive linear cascade (the links from A to B and from B to C are all positive). Note that the criteria of model selection used in our method are stricter than that of Hornung and Barkai [3]. For example, the biological circuit shown in Figure 4(a) does not satisfy the requirement for buffering propagated noise without a reduction in sensitivity in our analysis, while this biological circuit is competent for buffering noise while maintaining sensitivity in [3]. This is mainly due to two reasons. First, we employ the same group parameters to compare the candidate biological model with the linear cascade model. Second, the distance function in our method is much more rigorous.

Analysis of these eighty-one biological circuits is shown in Figure 4(e). There are twenty-four three-node biological circuits without positive feedback falling into the green area and these twenty-four biological networks are not competent for buffering noise while maintaining sensitivity. Thirty-three biological circuits with positive feedback (falling into gray area) fail in buffering noise while maintaining sensitivity. We find that there are three basic biological motifs that are indispensable for a biological circuit to be qualified for buffering noise without a reduction in sensitivity. The first basic motif is a self-positive feedback with a repressor, as shown in Figure 4(b), and we call it “Motif 1.” The second one is an indirect positive feedback with a repressor as shown in Figure 4(c), and we call it “Motif 2.” The third basic motif is an indirect positive feedback though a self-repressing node (Figure 4(d)), and we call it “Motif 3.” The biological circuits that behave well in buffering noise while maintaining sensitivity contain at least one of the three basic motifs. From Figure 4(e), we can find that there are eight biological circuits containing “Motif 1,” fifteen biological circuits containing “Motif 2,” nine biological circuits containing “Motif 3,” falling into the yellow area, dark red area, and luminous red area, respectively. There are three biological circuits with the combination of “Motif 1” and “Motif 2,” five biological circuits with the combination of “Motif 2” and “Motif 3,” and one biological circuit with the combination of “Motif 1,” “Motif 2,” and “Motif 3.” Therefore, there are forty-one biological circuits that are qualified for buffering noise while maintaining sensitivity. To better understand the mechanism underlying the ability of biological circuits to buffer noise for a given sensitivity, we analyzed a real noise-buffering biological circuit in the next section.

### 3.1. A Biological Example of Buffering Noise without a Reduction in Sensitivity

**Figure 5:**
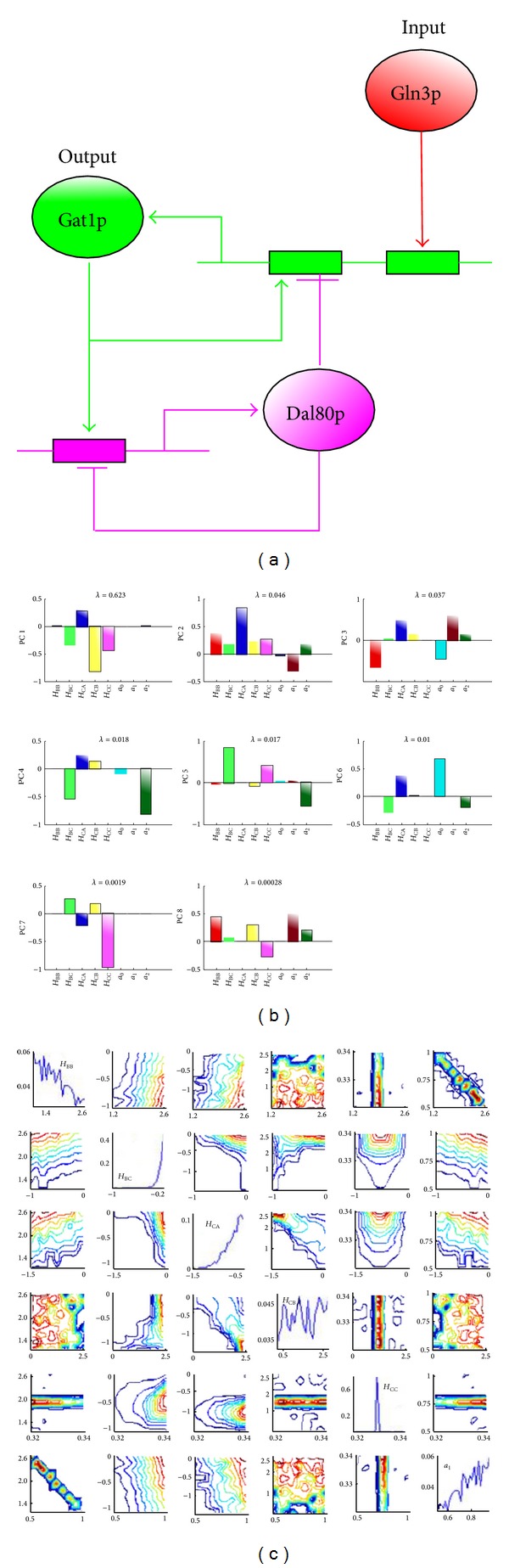
(a) In the yeast nitrogen catabolite repression system, the transcription factor Gat1p is activated in response to Gln3p. It can then activate its own transcription, as well as Dal80p, which binds to the same sequences as Gat1p and represses transcription. (b) Principal component analysis of the posterior distribution for model 6. PC1 is the first principal component, while PC8 describes the direction of least variance and therefore the most sensitive parameters. *a*_0_, *a*_1_, and *a*_2_ are the degradation constants for components A, B, and C, respectively. *λ* indicates the eigenvalue of variance covariance matrix. (c) Parameter posterior distribution for Model 6. *a*_1_ is the normalized degradation time scale for Da.

A well-studied biological example of buffering noise for a given sensitivity is the network involved in nitrogen homeostasis in yeast [3, 30, 31]. Here, a transcription factor (Gat1p), which is activated by nuclear Gln3p, feeds back to enhance its own transcription and in addition induces a transcriptional repressor (Dal80p) that competes with Gat1p for the same DNA binding sites (as shown in Figure 5(a)). This competition effectively weakens the positive feedback and ensures stability. Note that the sixth biological circuit in Figure 2(a) is the simplified form of this real biological case.

Denoting the input signal to the system by *n*_A_, the output Gat1p by *n*_C_, and the repressor Dal80p by *n*_B_, the system can be modeled by the following differential equations [30]:

(13) $$\begin{array}{c} \frac{dn_{\text{B}}}{dt}=\beta_{1}\frac{n_{\text{C}}}{k_{\text{BC}}+n_{\text{C}}+k_{\text{BC}}{\left(n_{\text{B}}/k_{\text{BB}}\right)}^{2}}-a_{1}n_{\text{B}},\\ \frac{dn_{\text{C}}}{dt}=\beta_{2}\frac{n_{\text{A}}n_{\text{C}}}{k_{\text{CC}}+n_{\text{C}}+k_{\text{CC}}\left(n_{\text{B}}/k_{\text{CB}}\right)}-a_{2}n_{\text{C}}, \end{array}$$

where *β*_*i*_ and *a*_*i*_ denote the maximum transcription rate and degradation constants. The *k*_*ij*_ (*i*, *j* = B, C) coefficients in the protein production terms are dissociation constants.

To operate as a sensitive noise buffer, this model must work in a regime where all interactions are unsaturated [3, 30]. Therefore, all the binding constants of the repressor, *k*_*i*B_, must be small, while all binding constants of the activator, *k*_*i*C_, must be large. Besides, the repressor, Dal80p, often responds more rapidly than Gat1p and Gln3p [3, 30] and it can be assumed to be at quasi-steady state. Taking (13) and the above conditions into consideration, we obtain

(14) $$\begin{array}{c} \frac{dn_{\text{C}}}{dt}=\beta_{2}n_{\text{A}}\frac{k_{\text{CB}}}{k_{\text{CC}}}\left(\frac{\beta_{1}}{\tau_{1}}\frac{k_{\text{BB}}^{2}}{k_{\text{BC}}}\right){n_{\text{C}}}^{2/3}-\tau_{2}n_{\text{C}}. \end{array}$$

Based on the definition of elasticity, the power law dependence of the transcription rate on *n*_C_ results in an almost-constant elasticity *H*_CC_ = 1/3 [3]. Hence, this network can buffer noise and maintain sensitivity for a large range of concentrations at which it remains unsaturated. Detailed simulations confirm that this biological circuit can indeed buffer propagated noise without a reduction in sensitivity. It ranks sixth in 432 biological circuits, as shown in Figure 2(a). The posterior distribution obtained via approximate Bayesian computation also confirms the range of elasticity *H*_CC_, as shown in Figure 5(c).

From Figure 5(c), we can see that the self-reaction flux elasticity, *H*_BB_, and the degradation time scale, *a*_1_, are correlated. To better understand the relations among the parameters, we employ the principal component analysis (PCA) to analyze posteriors of the parameters, as shown in Figure 5(b). In perspective of parameter sensitivity, we can see that *H*_CA_ and *H*_BC_ are less important.

## 4. Discussions

In this paper, the biological circuits qualifying for buffering noise without a reduction in sensitivity are studied. In many systems such as the sensing of temperature, nutrient levels, and ligand concentration, the signal is interpreted as a long-term change in the input, whereas noise is characterized by rapid stochastic fluctuations. In this study, we focus on this particular class of systems. Besides, in this work we assume that the noise is from the input signal and did not take into account intrinsic noise that arises from translational bursts [32–34]. Biological circuits involving intrinsic noise will be discussed in future work.

Paulsson's research [27] revealed that the noise propagation depends on two factors: the sensitivity to changes in input on the one hand and the averaging time [27, 28] on the other hand. Positive feedback is a central motif allowing for the buffering of propagated noise while maintaining sensitivity [3], since it can delay the kinetics and therefore increase the averaging time, leading to attenuation of propagated noise. In fact, we can view the feedback modules as low-pass frequency filters [10, 35, 36] and define a critical frequency above which fluctuations are eliminated and then negative feedback increases this critical frequency, allowing more propagated noise to pass, whereas positive feedback decreases this frequency and, thus, reduces the amount of noise.

Genetic circuits can implement elaborated tasks of amplitude or frequency signal detection. Guantes et al. considered signals acting on a two-component module to analyze what type of constraints the module could experience in the performance of these tasks and how they were affected by molecular noise [11]. Their work focused on the limits imposed by circuit structure on its characteristic stimulus response, the functional plasticity of coherent feed-forward loops, and the seemingly paradoxical advantage of improving signal detection with noisy circuit components. In the current study, we developed a new design framework for biological circuits selection. Our approach allowed us to exploit methods from Bayesian statistics, including efficient exploration of models spaces and high-dimensional parameter spaces and the ability to rank models with respect to their ability to maintain signaling sensitivity while minimizing noise propagation. We ranked the 432 three-node biological circuits based on their abilities to buffer noise while maintaining sensitivity. Our method has advantages over traditional design approaches in that the modeling and model evaluation/characterization is incorporated directly into the design stage. The statistical nature of the proposed method has many attractive features including the handling of stochastic systems, the ability to perform model selection, and the handling of parameter uncertainty in a well-defined manner.

In this work, the design problem can be divided into two steps: first selecting the network structure and then designing the model parameters. Approximate Bayesian computation (ABC) has become an essential tool for model design, especially for designing the complex stochastic models. However, Robert et al. [37] argue the lack of confidence in ABC model choice, since the algorithm involves an unknown loss of information induced by the use of insufficient summary statistics. The approximation error of the posterior probabilities of the models under comparison may thus be unrelated to the computational effort spent in running an ABC algorithm. Nevertheless, it is not a concern in this work, since we use the full data set with no summary and define the posterior distributions through the desired system outputs.
